# Summary of Medical Science

**Published:** 1861-07

**Authors:** 


ART. X.—*Summary of Medical Science.* Edited by WALTER S. WELLS, M.D., compiler of Epitome of Braithwaite's Retrospect, etc. Part i., April, 1861. New York, Charles T. Evans.

WE are not of those who think it a matter of consequence that the large mass of papers contributed to American and Transatlantic medical journals should be preserved in any other form for reference than that in which they first saw the light. Too often it happens that a practitioner, moving in a limited circle of information, or perhaps an extensive circle of ignorance, never meeting with anything out of the usual run of cases, nor prescribing anything out of the ordinary routine practice of the books, meets with something that puzzles him, and induces him to rub a little of the rust off the surface of his medical reading. He can find nothing in his books to elucidate the matter; he has nothing to refer to in the medical journal way, but what is about a quarter of a century old, and no fellow colaborer in the same cause in his confined atmosphere, who is not equally unfamiliar with recent literature. The next thing we hear of the case is its appearance in the "—— Medical Journal," unless the editor recognizes it as an old acquaintance of his younger days, and rejects it.

Occasionally, by a careful process of selection, something brilliant, sparkling in the midst of a mass of comparatively unimportant trifles, may be found worth preserving. He who knows how to sift the materials placed before him, and to distinguish the truly useful from the really valueless, is the best fitted to accomplish the task of compiling a "Summary," "Retrospect," "Abstract," or whatever else we may call it.

The cases, abstracts of which are contained in Dr. Wells's "Summary," are mainly derived from foreign medical periodicals. Those quoted from American journals are generally gleaned from the same source; and but few American observations are recorded, although the object of the work, as announced in the preface, is "to present to the profession a semi-annual digest of the essentially practical articles embraced in the principal European and American medical journals." The compiler thinks, perhaps, it is wiser to furnish to Cisatlantic readers chiefly the abstracts of cases which are not so readily accessible as those published at home. Dr. Wells would have received, we have no doubt, just as much credit as a compiler, had he republished in his "Summary" a larger number of the abstracts carefully prepared in our medical journals, of both foreign and American cases of interest.

Every collector of abstracts labors under this disadvantage, that no medical reader is ever perfectly satisfied with the selection presented to him, but almost involuntarily entertains the idea that, had the task been allotted to *him*, he would have been more successful in his choice of subjects. We acknowledge, from our own experience, that the act of selection is not always easy. We miss from the contents of Dr. Wells's work a large number of the valuable cases which have been, by competent *abstractors*, if we may employ such a term, deemed worthy of a place in the department assigned to abstracts in this and other journals. Their presence would have given additional merit to the "Summary," while their absence detracts from its completeness.

The abstracts, however, are well done, though it is not easy, from the arrangement, to tell whether they are made by Dr. Wells and others from the journals at their disposal, or whether they occur as abstracts in the periodicals themselves. Thus, in one or two places we notice that the North American Medico-Chirurgical Review is quoted as the authority for cases, as if the compilers of the "Summary" had themselves made the abstracts from that journal, whereas they had been carefully prepared for the latter from other authorities. To those who are fond of studying the medical history of a past year in its details, the work before us will possess points of interest and of satisfactory reference.

